# The changing face of floodplains in the Mississippi River Basin detected by a 60-year land use change dataset

**DOI:** 10.1038/s41597-021-01048-w

**Published:** 2021-10-15

**Authors:** Adnan Rajib, Qianjin Zheng, Heather E. Golden, Qiusheng Wu, Charles R. Lane, Jay R. Christensen, Ryan R. Morrison, Antonio Annis, Fernando Nardi

**Affiliations:** 1 Department of Environmental Engineering, Texas A&M University, Kingsville, Texas USA; 2 U.S. Environmental Protection Agency, Office of Research and Development, Cincinnati, Ohio USA; 3 Department of Geography, University of Tennessee, Knoxville, Tennessee USA; 4 U.S. Environmental Protection Agency, Office of Research and Development, Athens, Georgia USA; 5 Department of Civil and Environmental Engineering, Colorado State University, Fort Collins, Colorado USA; 6 Water Resources Research and Documentation Center, University for Foreigners of Perugia, Perugia, Italy; 7 Institute of Environment and College of Arts, Sciences & Education, Florida International University, Miami, Florida USA

**Keywords:** Ecosystem services, Hydrology, Sustainability

## Abstract

Floodplains provide essential ecosystem functions, yet >80% of European and North American floodplains are substantially modified. Despite floodplain changes over the past century, comprehensive, long-term land use change data within large river basin floodplains are limited. Long-term land use data can be used to quantify floodplain functions and provide spatially explicit information for management, restoration, and flood-risk mitigation. We present a comprehensive dataset quantifying floodplain land use change along the 3.3 million km^2^ Mississippi River Basin (MRB) covering 60 years (1941–2000) at 250-m resolution. We developed four unique products as part of this work, a(n): (i) Google Earth Engine interactive map visualization interface, (ii) Python code that runs in any internet browser, (iii) online tutorial with visualizations facilitating classroom code application, and (iv) instructional video demonstrating code application and database reproduction. Our data show that MRB’s natural floodplain ecosystems have been substantially altered to agricultural and developed land uses. These products will support MRB resilience and sustainability goals by advancing data-driven decision making on floodplain restoration, buyout, and conservation scenarios.

## Abstract (summary)

**Table:** 

Measurement(s)	land use process
Technology Type(s)	Geographic Information System
Factor Type(s)	floodplain land use change
Sample Characteristic - Environment	flood plain
Sample Characteristic - Location	Mississippi River Basin

Machine-accessible metadata file describing the reported data: 10.6084/m9.figshare.14804514

## Background & Summary

Riverine floodplains are vital and productive ecosystems that provide essential biological, geomorphic, and hydrologic functions^1,2^. Services provided by floodplains – including regulation of disturbances (e.g., flood attenuation), water supply, and waste treatment – are valued at approximately US$1.5 × 10^12^ yr^-1^ globally (in 2007 US$)^3^. Yet floodplains are continually threatened by human development and encroachment, including loss of floodplain-river connectivity due to channelization and levee construction^4^, which exacerbates habitat loss^5^ and hydrologic alteration^6^.

Human modifications to floodplains include changes in land use from activities such as urbanization, agriculture, industry, and mining^7^. For instance, approximately 80–90% of floodplains across Europe have been intensively cultivated^8^, and 90% of floodplains in North America are non-functional due to cultivation^9^. New developments in floodplains expose an increased population in the United States to flooding^10^, and even a 1% chance of flooding can cause losses exceeding $78 billion per year in the US^11^.

Flood-risk management efforts of the previous century have focused on minimizing flood impacts on humans through large and expensive infrastructure projects^12^ at the expense of floodplain ecosystem health and resilience. However, programs such as floodplain buyouts and conservation can produce co-benefits for economies and floodplain ecosystems^13^. Yet they require a comprehensive understanding of the history of floodplain changes along the full river continuum to ensure sustainable and effective floodplain and flood-risk management^14,15^.

Despite the human-induced changes in floodplains over the past century, comprehensive data of long-term land use change within floodplains of large river basins are limited^16^. A recent large-scale study assessed floodplain conditions across England from 1900 to 2015 using land use data^17^. Others have focused on changes across smaller geographic expanses and shorter time scales, such as floodplain losses in Dhaka, Bangladesh^18^ and Kumasi, Ghana^19^. No studies, to our knowledge, have integrated long-term ( > 30 year) data to examine changes in floodplain land use across a large river basin. Data of long-term and large-scale floodplain land use are required (1) to effectively quantify floodplain functions and development trajectories, and (2) for a holistic perspective on the future of floodplain management and restoration and concomitantly flood-risk mitigation.

**Fig. 1:**
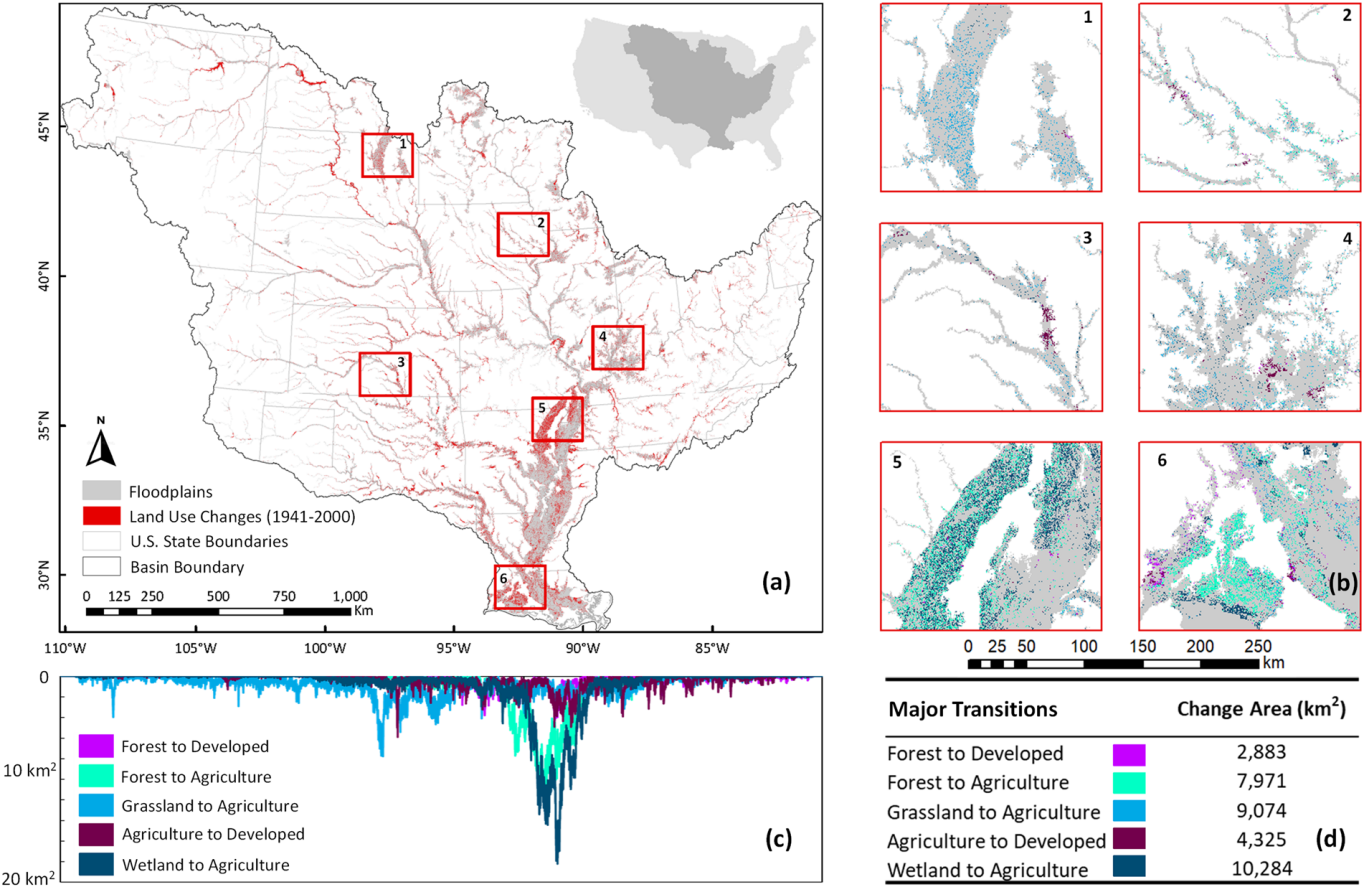
Land use change in the Mississippi River Basin (MRB) floodplains between 1941 and 2000. (**a**) The “change” in this map is defined as the non-uniqueness of individual land use grid cells between the two end years. (**b**) The maps 1–6 correspond to six objectively chosen domains across different geophysical settings and stream orders in South Dakota (1), Iowa (2), Kansas (3), Indiana (4), Arkansas (5), and Louisiana (6). These maps show five major land transitions in MRB floodplains that are potentially irreversible (e.g., wetland → agriculture), posing negative impacts on floodplain ecohydrology and resilience. Plot (**c**) graphically shows how the five major potentially irreversible land transitions vary along the latitude at every 250-m horizontal resolution. Plot (**d**) summarizes the areal extent (km^2^) of change between 1941 and 2000. The changes demonstrated in Fig. 1 can be further visualized at this interactive map interface: https://gishub.org/mrb-floodplain.

**Fig. 2:**
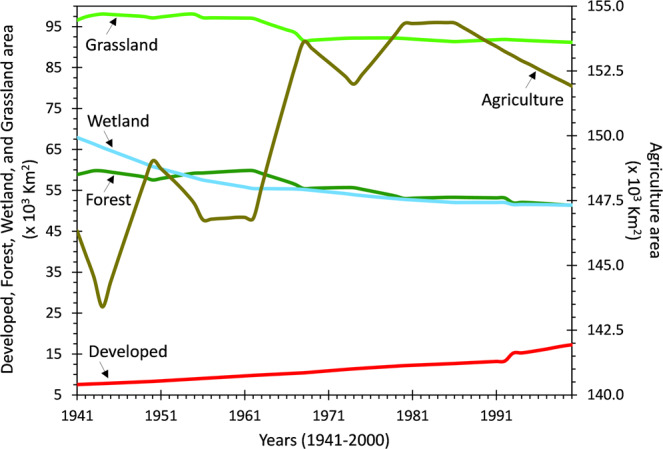
Time series graphs showing 60 years (1941–2000) of continuous changes in different land use classes. While values in this figure are aggregated for the entire Mississippi River Basin floodplains, time-series of changes for each of the six domains shown in Fig. 1 are separately plotted in Supplementary Fig. 1.

Here, we present the first available dataset to our knowledge that quantifies land use change along the floodplains of the Mississippi River Basin (MRB) covering 60 years (1941–2000) at 250-m resolution. The MRB is the fourth largest river basin in the world (3,288,000 km^2^) comprising 41% of the United States and draining into the Gulf of Mexico, an area with an annually expanding and contracting hypoxic zone resulting from basin-wide over-enrichment of nutrients^20^. The basin represents one of the most engineered systems in the world, and includes a complex web of dams, levees, floodplains, and dikes. This new dataset reveals the heterogenous spatial extent of land use transitions in MRB floodplains. The floodplains have transitioned from natural ecosystems to predominantly agricultural land use (e.g., more than 10,000 km^2^ of wetland loss due to agricultural expansion; Fig. 1). Developed land use within floodplain has also steadily increased (Fig. 2). These irreversible transitions in floodplain composition reduce storage^21^ and conveyance^22^ of natural flow, amplify flood risks posed by climate change^23,24^, and hinder both ecosystems and human well-being^25^.

To maximize the reuse of this dataset, we also include four unique products: (i) a Google Earth Engine interactive interface mapping MRB floodplain land use change over 60 years, (ii) a Google-based Python code that runs in any internet browser, (iii) an online tutorial with visualizations facilitating classroom application of the code, and (iv) an instructional video showing how to run the code and partially reproduce the dataset. We share all data through HydroShare: 10.4211/hs.41a3a9a9d8e54cc68f131b9a9c6c8c54.

The 60 years of spatially explicit floodplain land use change data produced herein are usable for flood-risk and nutrient management and research across the 31 US states that drain the MRB. A recent strategic plan of the Upper Mississippi River Restoration partnership, representing 0.5 million km^2^ of the MRB, envisions “a healthier and more resilient Upper Mississippi River ecosystem that sustains the river’s multiple use”^26^. This data will help achieve these goals by providing foundational information for data-driven decision making on floodplain restoration, buyouts, and conservation. Importantly, the data and associated materials can be the template for developing similar datasets for other river basins across the globe.

## Methods

### Input Data Sources

We derived the 60-year MRB floodplain land use change dataset from two input data sources: (i) the high-resolution global floodplain extent dataset GFPLAIN250m developed by Nardi *et al*.^27^, and (ii) the annual continental United States land use data developed by USGS^28,29^. GFPLAIN250m is based on a geomorphic analysis of the Digital Terrain Model (DTM) identifying riparian areas underlying maximum flood levels. The GFPLAIN algorithm estimates distributed flood energy gradients, at the river basin scale, with a simplified hydrologic model that assigns every channel cell a maximum flood depth using the drainage area as a scaling variable^30,31^. Conceptually, this algorithm dissects floodplains from surrounding hillslopes as those low-lying landscape features that have been naturally shaped by accumulated geomorphic effects of past flood events. Therefore, the GFPLAIN250m dataset is built on the principle that a flood-prone area is implicitly contained in the DTM, decoupling the floodplain extent zoning from the need to preliminarily define a design flood event. The outcome is a DTM-based morphometric indexing of floodplain domains rather than a simulation associated to a specific return period, e.g., 100-year^32^. The dataset is publicly available at 250-m spatial resolution gridded GeoTIFF format, with coordinates set by World Geodetic System 1984 (WGS84).

The USGS land use data is based on a spatially explicit modeling framework which reconstructed a temporally continuous land use from widely acknowledged baselines including the National Land Cover Databases (NLCD)^33–35^, more than 100 years of agricultural census information^36^, and three decades of representative satellite imagery^37^. The dataset is publicly available at 250-m spatial resolution in gridded GeoTIFF format, with coordinates set by USA Contiguous Albers Equal Area Conic USGS version projected system. This land use dataset is divided into two parts: a 14-class historical land use for each year from 1938 to 1992^28^ and a 17-class recent land use for each year from 1992 to 2005^29^. We included the land use from 1941 to 2000 in our approach to develop the 60-year MRB floodplain land use change dataset.

### Procedure

**Fig. 3:**
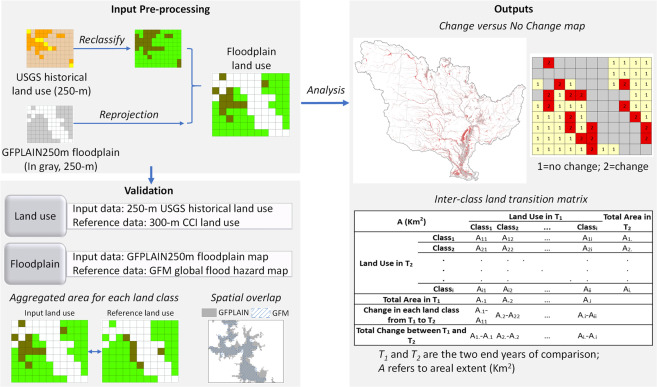
Schematic of the methodological design.

Our methodology followed six consecutive steps (Fig. 3): (i) reprojection of floodplain and land use data to a consistent coordinate system, (ii) land use reclassification, (iii) extraction of floodplain land use, (iv) land use change detection, (v) formation of inter-class land transition matrix, and (vi) technical validation. These steps are discussed in detail below. All the associated tasks were performed in ArcGIS 10.5 and ENVI 5.1 geospatial analysis platforms.

**Fig. 4:**
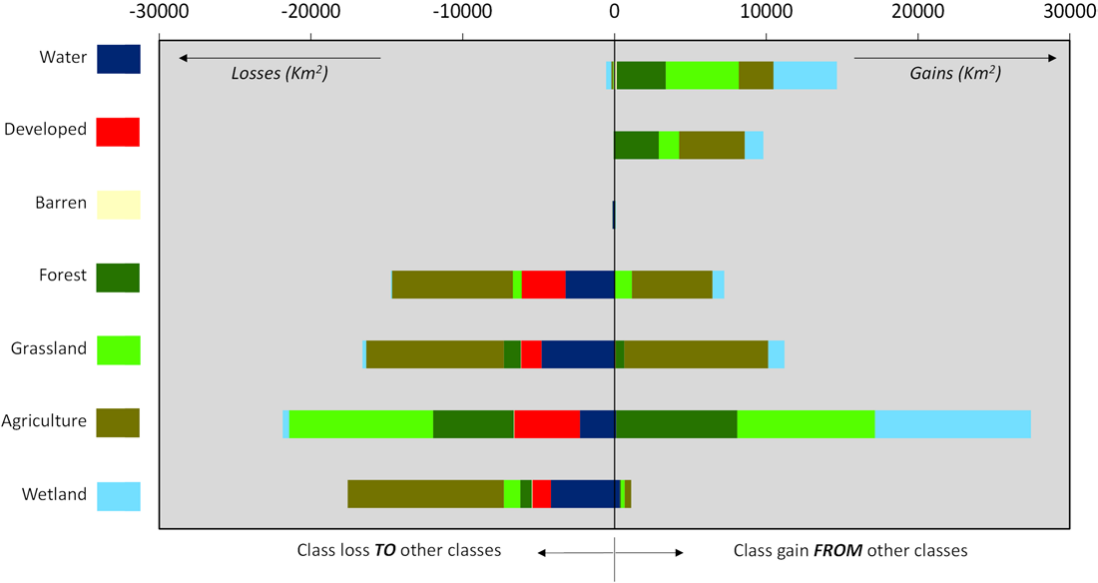
Inter-class land loss versus gain between 1941 and 2000 for the entire MRB floodplains.

(i) *Reprojection of coordinate systems*: The two primary inputs used in our approach, i.e., the global floodplain and annual USGS land use, were originally developed in two different coordinate systems. Because non-identical coordinate systems across corresponding datasets induce positional error in floodplain geospatial analysis especially across large river basins^38,39^, we reprojected the coordinate system of the global floodplain to that of the USGS land use such that the two datasets become interoperable.
(ii) *Land use reclassification*: Commonly used land use datasets follow classification schemes (i.e., categorizing the intended purpose of a landscape parcel^40^) with multiple levels of nested hierarchy^41^. While a detailed land use classification offers critical insights to environmental monitoring and restoration research^42,43^, the large semantic variability of land use classifications across disciplines often complicates their practical applications^44^. To allow easy integration of our land use change dataset with cross-disciplinary research and decision-making tools, we simplified the original classification scheme of the USGS land use data to produce seven generic classes. The new land use classes included: 1) open water, 2) developed area, 3) barren land, 4) forest, 5) grassland, 6) agriculture, and 7) wetland.
(iii) *Extraction of 60-year floodplain land use*: We used the MRB boundary polygon as a mask and clipped the MRB portion of the global floodplain (hereafter, the MRB floodplain). Following the same approach in a subsequent step, we used the MRB floodplain as a mask on the USGS land use and extracted a series of floodplain land use maps for each of the years from 1941 to 2000. We then calculated the areal extents of different land use classes by multiplying their corresponding total number of grid-cells with the spatial resolution of a single grid-cell (250*250 m^2^), thus creating 60-year time-series of floodplain land use in the MRB (e.g., Fig. 2 and Supplementary Fig. 1). Since the USGS land use dataset was developed only for the continental United States, the small upstream portion of the MRB that drains two Canadian provinces (~1% of the basin’s drainage area) was excluded from our analysis.
(iv) *Land use change detection between two end-years (*1*941 and* 2*000)*: We applied a statistical approach to detect the difference/non-uniqueness in land use between the two end-years of comparison (i.e., 1941 and 2000). Specifically, we calculated the number of unique grid-cell values between the two land use maps on a cell-by-cell basis. The outcome was a new map with only two possible values in the grid-cells. Value “1” indicated one unique value of a target grid-cell across the two input land use maps, meaning “no change” of land use between two points in time. Conversely, value “2” indicated that a target grid-cell had two non-unique values and hence a “change” of land use between two points in time.
(v) *Formation of inter-class land transition matrix*: To demonstrate the “nature of change”^45^ in the MRB floodplains, we quantified how the land use therein transitioned from one class to the other(s) between two end-years (1941 and 2000). We conducted this task using a widely acknowledged approach called *Transition Matrix* Analysis^33,46–53^. Table 1 schematically shows the resultant transition matrix across the seven land use classes of the MRB floodplains. Here, *T*_1_ and *T*_2_ respectively indicate the two end-years of comparison, while *A*_*ij*_ is the areal extent [L^2^] that transitioned from class *i* at the initial year to class *j* at the final year. The last row in the transition matrix represents the net gain or loss of areal extent between *T*_*1*_ and *T*_*2*_ in every land use class. The inter-class land use transitions in the MRB floodplains between 1941 and 2000 are graphically presented in Fig. 4, while the corresponding calculations are provided in Supplementary Table 1.

**Table 1:** Schematic of the inter-class land transition matrix.

		Area in year T_1_^*^							Total area in T_2_^**^
		Class_1_	Class_2_	Class_3_	Class_4_	Class_5_	Class_6_	Class_7_	
**Area in year T**_2_^*^	**Class**_**1**_	A_11_	A_12_	A_13_	A_14_	A_15_	A_16_	A_17_	A_1_.
	**Class**_**2**_	A_21_	A_22_	A_23_	A_24_	A_25_	A_26_	A_27_	A_2_.
	**Class**_**3**_	A_31_	A_32_	A_33_	A_34_	A_35_	A_36_	A_37_	A_3_.
	**Class**_**4**_	A_41_	A_42_	A_43_	A_44_	A_45_	A_46_	A_47_	A_4_.
	**Class**_**5**_	A_51_	A_52_	A_53_	A_54_	A_55_	A_56_	A_57_	A_5_.
	**Class**_**6**_	A_61_	A_62_	A_63_	A_64_	A_65_	A_66_	A_67_	A_6_.
	**Class**_**7**_	A_71_	A_72_	A_73_	A_74_	A_75_	A_76_	A_77_	A_7_.
**Total area in T**_1_^***^		A._1_	A._2_	A._3_	A._4_	A._5_	A._6_	A._7_	
**Change in each land class between T**_**1**_ **to T**_**2**_		A._1_-A_11_	A._2_-A_22_	A._3_-A_33_	A._4_-A_44_	A._5_-A_55_	A._6_-A_66_	A._7_-A_77_	
**Total change between T**_**1**_ **and T**_**2**_		A_1_.-A._1_	A_2_.-A._2_	A_3_.-A._3_	A_4_.-A._4_	A_5_.-A._5_	A_6_.-A._6_	A_7_.-A._7_	

*T_1_ and T_2_ are the two end-years of comparison; A refers to areal extent for a land use class (Km^2^); diagonal cells in the table indicate “no change” between T_1_ and T_2_; **Sum of values in a row; ***Sum of values in a column.

## Data Reco rds

The MRB floodplain land use change dataset is made available through an open-access geospatial data sharing platform HydroShare. Our archive also includes all corresponding input data, intermediate calculations, and supporting information. Tables 2 and 3 below provide an overview of the file contents. The entire archive can be downloaded as a single zip file from this web address: 10.4211/hs.41a3a9a9d8e54cc68f131b9a9c6c8c54^54^.

**Table 2:** Input dataset file descriptions.

Folder Name: Input Data				
ID	Subfolder/File Name	File Type	Content Description	Provenance
1	MRB_floodplain	GIS raster*	Riverine floodplain extents within the Mississippi River Basin (MRB), clipped from the GFPLAIN250m floodplain dataset • 250-m grid, GeoTIFF format • Albers Equal Area Conic projected coordinate	Nardi *et al*.^27^.
2	MRB_floodplain_LU	GIS raster*	Input land use dataset for change detection • Clipped for the MRB floodplain extent • Modified to have seven generic land use classes • One corresponding dataset for each of the years from 1941 to 2000	Sohl *et al*.^28,29^; Supplementary Table 2
3	Reference_LU	GIS raster*	Reference land use dataset used for validation purposes (see items #10 and 11 in Table 3) • Clipped for the MRB floodplain extent • Modified to have same land use classes as in item #2 • One corresponding dataset for each of the years from 1992 to 2000	European Space Agency^58^; Supplementary Table 3
4	Reference_floodplain	GIS raster	Riverine floodplain extents within the MRB, clipped from the European Commission Joint Research Centre Global Flood Maps (GFM) • 1000-m grid, GeoTIFF format	Dottori *et al*.^56^

Note: All GIS raster and shapefile datasets are in the Albers Equal Area Conic projected coordinate system. *The raster datasets are in GeoTIFF format at 250-m spatial resolution (except item # 4).

**Table 3:** Output dataset file descriptions.

Folder Name: Output Data				
ID	Subfolder/File Name	File Type	Content Description	Output Figure/Table
5	ChangeMap	GIS raster*	A map showing the changes in MRB floodplain land use between 1941 and 2000; “change” is defined as the non-uniqueness of individual land use grid-cells • Grid value 1 indicates “no change” and 2 indicates “change”	Fig. 1a
6	ClassTransitionMaps	GIS raster*; shapefile	A map showing major land use class-transitions in MRB floodplains between 1941 and 2000 • Five irreversible transitions (e.g., wetland → agriculture) that would negatively impact floodplain ecohydrology and resilience Also includes boundary polygons for six objectively chosen domains to facilitate focused assessments	Fig. 1b
7	ClassTransition_ LatLong	MS Excel	Total area (km^2^) for each of the five irreversible transitions mentioned in item #6 • Separate calculations along the latitudinal and longitudinal directions at every 250-m spacing	Fig. 1c
8	ClassTransitionMatrix	MS Excel	Inter-class transitions (km^2^) across all seven generic land use classes between 1941 and 2000; based on the schematic presented in Table 1	Supplementary Table 1; Fig. 1d; Fig. 4
9	Timeseries	MS Excel	Timeseries of total area (km^2^) for each of the seven generic land use classes • 60-year timeseries with corresponding values for every year from 1941 to 2000 • Seven datasets: the entire MRB floodplains and the six domains mentioned in item #6	Fig. 2; Supplementary Fig. 1
10	ValidationMaps	MS Excel; GIS Shapefile	Measures of fit comparing the input and reference floodplain extents (Table 2 ‒ items #1 and 4 respectively) across the MRB • An Excel file showing 8-digit Hydrologic Unit Codes (HUC) and corresponding TP and CSI values • HUC-8 boundary polygon shapefile	Fig. 5b,c
		GIS raster*	Validation maps showing the spatial consistency between input and reference land use datasets (Table 2 ‒ items #2 and 3 respectively) • 12 validation domains • Maps correspond to different years between 1992 and 2000 • File nomenclature: *x_y_z*; *x* = USGS (input) or Remotely sensed (reference) land use, *y* = US state where the validation domain is located; *z* = year	Fig. 6; Supplementary Figs. 2–4
11	ValidationResults	MS Excel	Total area (km^2^) for different land use classes at each of the 12 validation domains, calculated separately from the two contrasting land use datasets (item #10) • Includes calculation of correlations (R^2^) between two land use datasets	

Note: All GIS raster and shapefile datasets are in the Albers Equal Area Conic projected coordinate system. *The raster datasets are in GeoTIFF format at 250-m spatial resolution (except Table 2 item# 4).

## Technical Validation

To ensure the technical quality and reliability of our MRB floodplain land use change dataset, we validated both GFPLAIN250m floodplain and USGS land use datasets with respect to the best available references.

**Fig. 5:**
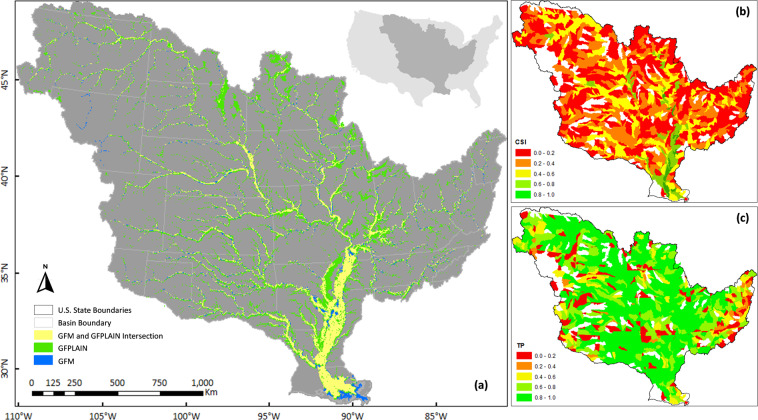
Consistency between GFPLAIN250m (input) and GFM (reference) floodplain extents. (**a**) Yellow areas show the intersection between the two datasets^27,56^. (**b**,**c**) Map of the Critical Success Index (CSI) and True Positive Rate (TP rate) for each Hydrologic Unit watershed where the two contrasting floodplain datasets are intersected. CSI = A/(A + B + C), TP rate = A/(A + C); here A, B, and C – the yellow, green, and blue areas in map (**a**), respectively, indicate the matches, areas covered by the GFPLAIN250m only, and areas covered by the GFM only.

Although the GFPLAIN250m floodplain^27^ has been previously validated across different scales^55^, we further compared it to the Global Flood Maps (GFM)^56^ across the MRB Hydrologic Unit Codes (HUCs). We used critical success index (CSI) and true positive rate (TP rate) metrics to confirm the spatial consistency between GFPLAIN250m and GFM floodplain extents (Fig. 5). When compared to GFM, GFPLAIN tends to produce larger floodplain delineations in the headwater regions of the MRB (Fig. 5a). This was also evident in Fig. 5b,which showed lower CSI values in the headwater HUCs. Values of CSI were within acceptable ranges demonstrated by previous studies^27,57^, however. TP rates (Fig. 5c) were greater than 0.8 for most of the HUCs, indicating a high overlap between GFPLAIN250m and GFM datasets. These assessments demonstrate the applicability of using the GFPLAIN250m data to identify continuous floodplains.

**Fig. 6:**
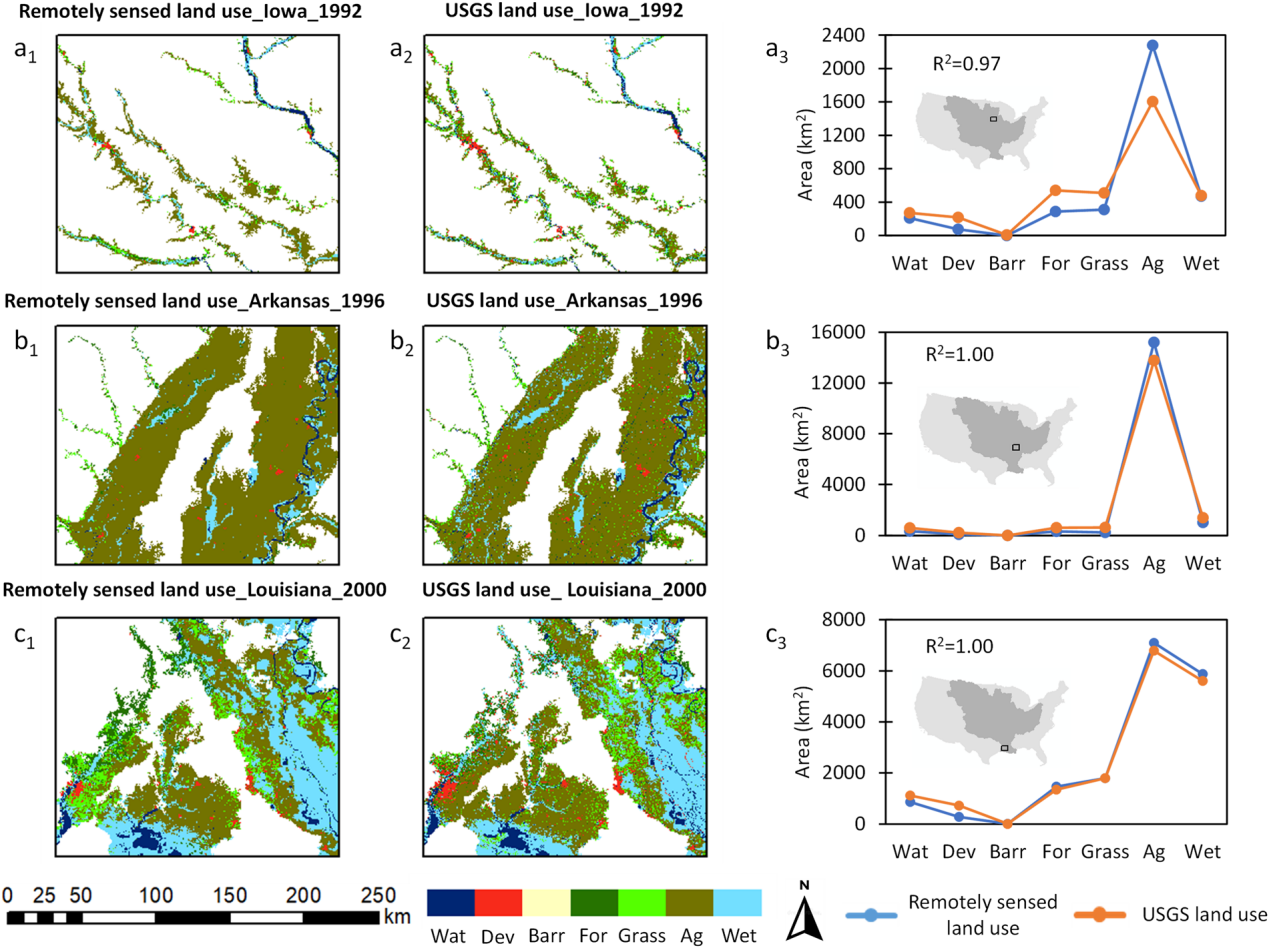
Spatial comparison between USGS (input) and remotely sensed (reference) land use datasets in three different years. The subplots (**a**–**c**) correspond to zoomed-in portions of the Mississippi River Basin floodplains in Iowa, Arkansas, and Louisiana, respectively. a_1_, b_1_, c_1_ show ESA’s Climate Change Initiative (CCI) land use maps based on satellite observations (hereafter, the remotely sensed land use)^58^, while a_2_, b_2_, c_2_ show the land use data obtained from USGS land modeling framework^28,29^. The remotely sensed land use (a_1_ − c_1_) was our reference to validate the spatial consistency of the USGS land use (a_2_ − c_2_; the input land use data in our methodology). Subplots a_3_ − c_3_ show the correlation between remotely sensed and USGS datasets across different land use classes within the given spatial domains. The generic land use classes include water, developed, barren, forest, grassland, agriculture, and wetland (abbreviated as Wat, Dev, Barr, For, Grass, Ag, and Wet, respectively).

We validated the USGS land use using the European Space Agency’s (ESA) Climate Change Initiative (CCI) data. The CCI data include a time-series of consistent global land use maps at 300-m spatial resolution on an annual basis from 1992 to 2019^58^. These global land use maps were derived from multiple satellite data sources, including Envisat Medium Resolution Imaging Spectrometer (MERIS) (2003–2012), Advanced Very High Resolution Radiometer (AVHRR) (1992–1999), SPOT-VGT (1999–2013), and PROBA-V (2013–2015)^59^ (hereafter, we refer CCI land use as the *remotely sensed land use* for simplicity). In contrast, the 250-m spatial resolution USGS land use maps were based on a hindcast modeling (1938–2005), derived from the NLCD, Landsat satellite, and county-level agricultural census. We chose the CCI/remotely sensed dataset as the reference because it was developed by a different agency with different data sources, which ensured an independent validation of our land use change estimates (see Fig. 6).

The USGS land use contains 17 classes (Supplementary Table 2), whereas the remotely sensed land use contains 37 classes (Supplementary Table 3). To make these two datasets comparable, we reclassified them into seven generic classes, including open water, developed area, barren land, forest, grassland, agriculture, and wetland (see Supplementary Tables 2, 3). In addition, we reprojected the CCI coordinates from World Geodetic System 84 (WGS84) to USA Contiguous Albers Equal Area Conic projected system to enable a uniform comparison with the USGS data. After the reclassification and reprojection steps, we selected 12 sites to validate our MRB floodplain land use change dataset. These validation sites were chosen objectively to represent different geophysical settings across the MRB as well as different stream orders, including both major rivers and lower order tributaries. During the common period of data availability between USGS (input) and remotely sensed (reference) datasets, we conducted validations for the 12 selected sites from 1992 to 2000, with a different year randomly assigned to each validation site. It should be noted that the comparison was conducted at an aggregated level for each site. We did not evaluate the cell-by-cell correlations for two reasons. First, the two datasets were developed at two different spatial resolutions (250-m and 300-m). Second, the respective definitions of land classes are not identical across the two datasets, although we reclassified them to bring some degree of consistency. Three validation sites are shown in Fig. 6, and the other nine validation sites are shown in Supplementary Figs. 2–4.

The validation results for the 12 selected sites show high correlations between the USGS and remotely sensed data across all land use classes, with R^2^ ranging from 0.90 to 0.99. Agricultural land and grassland appear to show the largest discrepancy between the USGS and remotely sensed data among the seven land use classes, which is largely due to the potential inconsistency in respective land class definition schemes between these two datasets. The original USGS classification of hay/pasture was designated as grassland while in the remotely sensed dataset, there is no hay/pasture class but a cropland with herbaceous cover which was designated as agriculture in our simplified classifications (Supplementary Tables 2, 3). The other five land use classes are highly consistent across all validation sites. Overall, these validation results indicate that our input and accordingly our output datasets are sufficiently reliable.

## Usage Notes

To ensure that the MRB floodplain land use change dataset, relevant inputs, and underlying methodology are Findable, Accessible, Interoperable, and Reproducible (FAIR)^60^, we developed four software solutions and educational products (Table 4). These products, besides assisting other researchers with the reuse of our dataset, will also foster new research on floodplain resilience by allowing efficient analysis of floodplain land use change in any of the world’s major river basins.

**Table 4:** Software solutions and educational materials for enhancing the FAIR data properties.

	Product	Access Link and Intended Objectives
1	Web interface	https://gishub.org/mrb-floodplain A Google Earth Engine interface facilitating the interactive visualization of major land use class-transitions in MRB floodplains (see item #6 in Table 3).
2	Semi-automatic coding framework	https://colab.research.google.com/drive/1vmIaUCkL66CoTv4rNRIWpJXYXp4TlAKd?usp = sharing A ready-to-use python code that operates entirely in Google’s web-based high-performance programming platform called *Google Colaboratory*. • Allows users to reproduce the MRB floodplain land use change dataset (up to the item #5 listed in Table 3); users can run the code simply in a web browser without requiring to write any new code or setting up a programming environment in users’ local computers. • Not limited to change detection only; an end-to-end workflow that performs all data discovery, download, and pre-processing tasks in a semi-automatic manner. • Scalable to the floodplains in any of the world’s major river basins. In addition to the specific land use input^28,29^ used in our work to generate the MRB floodplain land use change dataset, the current version of our code can also assimilate land use input from two different sources: 30-m National Land Cover Database (United States)^33–35^ and 300-m Climate Change Initiative Land Cover Database (global)^58^.
3	Classroom tutorial	https://serc.carleton.edu/hydromodules/steps/241489.html A web-based tutorial offering step-by-step instructions to run the Google Colaboratory python code, partially reproduce the MRB floodplain land use change dataset, and perform offline visualization of the output dataset in ArcGIS desktop; built to fit classroom instruction modules.
4	Instructional video	https://youtu.be/wH0gif_y15A A You Tube video that shows how to run the Google Colaboratory python code following our web-based tutorial; facilitates more efficient reproduction of the dataset.

## Supplementary information

Supplementary Information

## Data Availability

The MRB floodplain land use change dataset is derived entirely through ArcGIS 10.5 and ENVI 5.1 geospatial analysis platforms (see *Methods* section for details). We developed additional open-access codes and visualization interfaces, however, to promote reproducibility and widespread application of the dataset. The python code is accessible at: https://colab.research.google.com/drive/1vmIaUCkL66CoTv4rNRIWpJXYXp4TlAKd?usp = sharing. The visualization interface is available online at: https://gishub.org/mrb-floodplain. See *Usage Notes* section for details.
