# Supplementary material for: The changing face of floodplains in the Mississippi River Basin detected by a 60-year land use change dataset
Source: Sci Data. 2021 Oct 15;8:271. doi: 10.1038/s41597-021-01048-w (PMC8520011; doi:10.1038/s41597-021-01048-w)
Supplement: Supplementary file 1 — Supplementary Information [file 41597_2021_1048_MOESM1_ESM.docx]

**Supplementary information**

**for**

**The Changing Face of Floodplains in the Mississippi River Basin**

**Detected by A 60-year Land Use Change Dataset**

Submitted to the *Scientific Data*

**Adnan Rajib^1^, Qianjin Zheng^1^, Heather E. Golden^2^, Qiusheng Wu^3^, Charles R. Lane^4^, Jay R. Christensen^2^, Ryan R. Morrison^5^, Antonio Annis^6^, Fernando Nardi^6,7^**

^1^ Department of Environmental Engineering, Texas A&M University, Kingsville, Texas, USA

^2^ U.S. Environmental Protection Agency, Office of Research and Development, Cincinnati, Ohio, USA

^3^Department of Geography, University of Tennessee, Knoxville, Tennessee, USA

^4^ U.S. Environmental Protection Agency, Office of Research and Development, Athens, Georgia, USA

^5^ Department of Civil and Environmental Engineering, Colorado State University, Fort Collins, Colorado, USA

^6^ Water Resources Research and Documentation Center, University for Foreigners of Perugia, Perugia, Italy

^7^ Institute of Environment and College of Arts, Sciences & Education, Florida International University, Miami, Florida, USA

Corresponding author: Adnan Rajib (adnan.rajib@tamuk.edu)

**Table of Contents**

[**Supplementary Tables** 3](#_Toc62585832)

[**Supplementary Table 1.** 3](#_Toc62585833)

[**Supplementary Table 2.** 4](#_Toc62585834)

[**Supplementary Table 3.** 5](#_Toc62585835)

[**Supplementary Figures** 6](#_Toc62585836)

[**Supplementary Figure 1.** 6](#_Toc62585837)

[**Supplementary Figure 2.** 7](#_Toc62585838)

[**Supplementary Figure 3.** 8](#_Toc62585839)

[**Supplementary Figure 4.** 9](#_Toc62585840)

[**References** 10](#_Toc62585841)

# **Supplementary Tables**

**Supplementary Table 1.** Inter-class transitions (km^2^) across seven generic land use classes between 1941 and 2000. The values shown here represent the aggregated estimates across the entire Mississippi River Basin floodplains. The structure of the inter-class transition matrix is schematically explained in Table 1.

|  | | **Land use in year 1941** **(Km^2^)** | | | | | | | **Total area in 2000** |
| --- | --- | --- | --- | --- | --- | --- | --- | --- | --- |
|  |  | **Water** | **Developed** | **Barren** | **Forest** | **Grassland** | **Agriculture** | **Wetland** |  |
| **Land use in year 2000** **(Km^2^)** | **Water** | 19,968 | 31 | 120 | 3,228 | 4,810 | 2,282 | 4,186 | 34,625 |
|  | **Developed** | 35 | 7,474 | 3 | 2,883 | 1,339 | 4,325 | 1,218 | 17,277 |
|  | **Barren** | 17 | 0 | 668 | 1 | 19 | 25 | 42 | 772 |
|  | **Forest** | 10 | 15 | 0 | 44,126 | 1,117 | 5,319 | 768 | 51,355 |
|  | **Grassland** | 45 | 14 | 8 | 579 | 79,975 | 9,477 | 1,078 | 91,176 |
|  | **Agriculture** | 107 | 8 | 2 | 7,971 | 9,074 | 124,471 | 10,284 | 151,916 |
|  | **Wetland** | 345 | 2 | 8 | 66 | 249 | 425 | 50,323 | 51,417 |
| **Total area in 1941** | | 20,527 | 7,543 | 809 | 58,853 | 96,584 | 146,324 | 67,899 |  |
| **Change in each land class between 1941 and 2000** | | 559 | 69 | 141 | 14,727 | 16,609 | 21,853 | 17,576 |  |
| **Difference** | | 14,098 | 9,734 | -37 | -7,498 | -5,408 | 5,592 | -16,481 |  |

**Supplementary Table 2.** Reclassification scheme of the USGS land use data^1,2^. The 17 original classes were reclassified to 7 generic classes. This reclassified land use data was the main input for change detection.

| **Original Class** | **New Class** | **Old Value** | **New Value** |
| --- | --- | --- | --- |
| Water | Water | 1 | 1 |
| Developed | Developed | 2 | 2 |
| Mechanically Disturbed National Forests | Developed | 3 | 2 |
| Mechanically Disturbed Other Public Lands | Developed | 4 | 2 |
| Mechanically Disturbed Other Public Lands | Developed | 5 | 2 |
| Mining | Developed | 6 | 2 |
| Barren | Barren | 7 | 3 |
| Deciduous Forest | Forest | 8 | 4 |
| Evergreen Forest | Forest | 9 | 4 |
| Mixed Forest | Forest | 10 | 4 |
| Grassland | Grassland | 11 | 5 |
| Shrubland | Grassland | 12 | 5 |
| Cropland | Agriculture | 13 | 6 |
| Hay/Pasture Land | Grassland | 14 | 5 |
| Herbaceous Wetland | Wetland | 15 | 7 |
| Woody Wetland | Wetland | 16 | 7 |
| Perennial Ice/Snow | Water | 17 | 1 |

**Supplementary Table 3.** Reclassification scheme of the remotely sensed land use data^3^. The 37 original classes were reclassified to 7 generic classes following Intergovernmental Panel on Climate Change (IPCC) guidelines^4^.

| **Original Class** | **New Class** | **Old Value** | **New Value** |
| --- | --- | --- | --- |
| Cropland | Agriculture | 10 | 6 |
| Herbaceous cover | Agriculture | 11 | 6 |
| Tree or shrub cover | Agriculture | 12 | 6 |
| Cropland irrigated or post-flooding | Agriculture | 20 | 6 |
| Mosaic cropland (>50%) /natural vegetation (tree shrub herbaceous cover) (<50%) | Agriculture | 30 | 6 |
| Mosaic natural vegetation (tree shrub herbaceous cover) (>50%) /cropland (<50%) | Agriculture | 40 | 6 |
| Tree cover broadleaved evergreen closed to open (>15%) | Forest | 50 | 4 |
| Tree cover broadleaved deciduous closed to open (>15%) | Forest | 60 | 4 |
| Tree cover broadleaved deciduous closed (>40%) | Forest | 61 | 4 |
| Tree cover broadleaved deciduous open (15-40%) | Forest | 62 | 4 |
| Tree cover needleleaved evergreen closed to open (>15%) | Forest | 70 | 4 |
| Tree cover needleleaved evergreen closed (>40%) | Forest | 71 | 4 |
| Tree cover needleleaved evergreen open (15-40%) | Forest | 72 | 4 |
| Tree cover needleleaved deciduous closed to open (>15%) | Forest | 80 | 4 |
| Tree cover needleleaved deciduous closed (>40%) | Forest | 81 | 4 |
| Tree cover needleleaved deciduous open (15-40%) | Forest | 82 | 4 |
| Tree cover mixed leaf type (broadleaved and needleleaved) | Forest | 90 | 4 |
| Mosaic tree and shrub (>50%) / herbaceous cover (<50%) | Forest | 100 | 4 |
| Mosaic herbaceous cover (>50%) / tree and shrub (<50%) | Grassland | 110 | 5 |
| Shrubland | Grassland | 120 | 5 |
| Shrubland evergreen | Grassland | 121 | 5 |
| Shrubland deciduous | Grassland | 122 | 5 |
| Grassland | Grassland | 130 | 5 |
| Lichens and mosses | Grassland | 140 | 5 |
| Sparse vegetation (tree shrub herbaceous cover) (<15%) | Grassland | 150 | 5 |
| Sparse tree (<15%) | Grassland | 151 | 5 |
| Sparse shrub (<15%) | Grassland | 152 | 5 |
| Sparse herbaceous cover (<15%) | Grassland | 153 | 5 |
| Tree cover flooded fresh or brakish water | Forest | 160 | 4 |
| Tree cover flooded saline water | Forest | 170 | 4 |
| Shrub or herbaceous cover flooded fresh/saline/brakish water | Wetland | 180 | 7 |
| Urban areas | Developed | 190 | 2 |
| Bare areas | Barren | 200 | 3 |
| Consolidated bare areas | Barren | 201 | 3 |
| Unconsolidated bare areas | Barren | 202 | 3 |
| Water bodies | Water | 210 | 1 |
| Permanent snow and ice | Water | 220 | 1 |

# **Supplementary Figures**


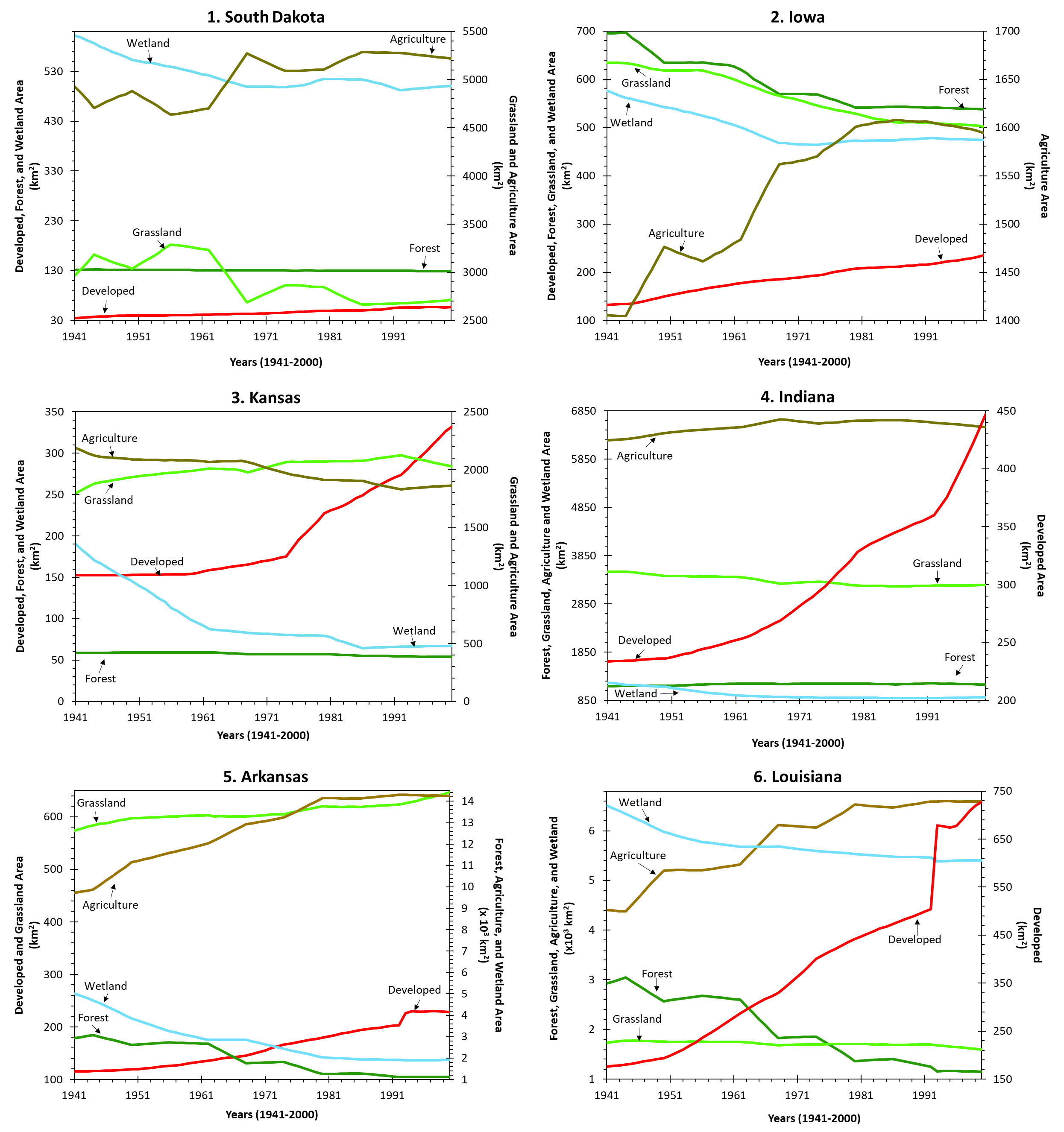


**Supplementary Figure 1.** Time-series graphs showing 60 years (1941-2000) of continuous changes in different land use classes across six objectively chosen domains (South Dakota, Iowa, Kansas, Indiana, Arkansas, and Louisiana). Figure 1 shows the location of these domains.

**
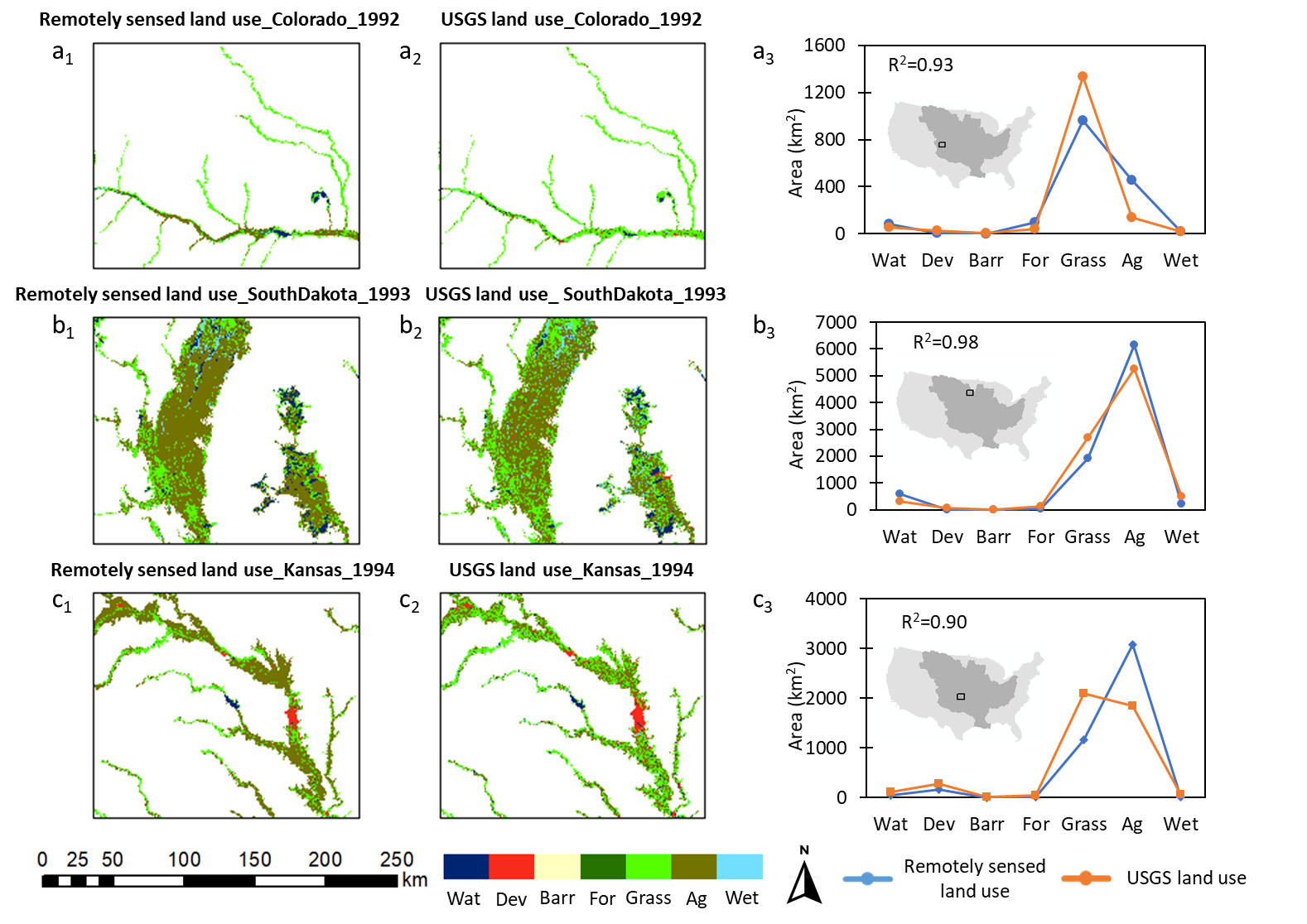
**

**Supplementary Figure 2.** Spatial comparison between input and reference land use datasets in three different years. The subplots a-c correspond to zoomed-in portions of the Mississippi River Basin floodplains in Colorado, South Dakota, and Kansas, respectively. a_1_, b_1_, c_1_ show ESA’s Climate Change Initiative (CCI) land use maps based on satellite observations (hereafter, the remotely sensed land use)^3,4^, while a_2_, b_2_, c_2_ show the land use data obtained from USGS land modeling framework^1,2^. The remotely sensed land use (a_1_ - c_1_) was our reference to validate the spatial consistency of the USGS land use (a_2_ – c_2_; the input land use data in our methodology). Subplots a_3_ - c_3_ show the correlation between remotely sensed and USGS datasets across different land use classes within a given spatial domain. The generic land use classes include water, developed, barren, forest, grassland, agriculture, and wetland (abbreviated as Wat, Dev, Barr, For, Grass, Ag, and Wet, respectively).


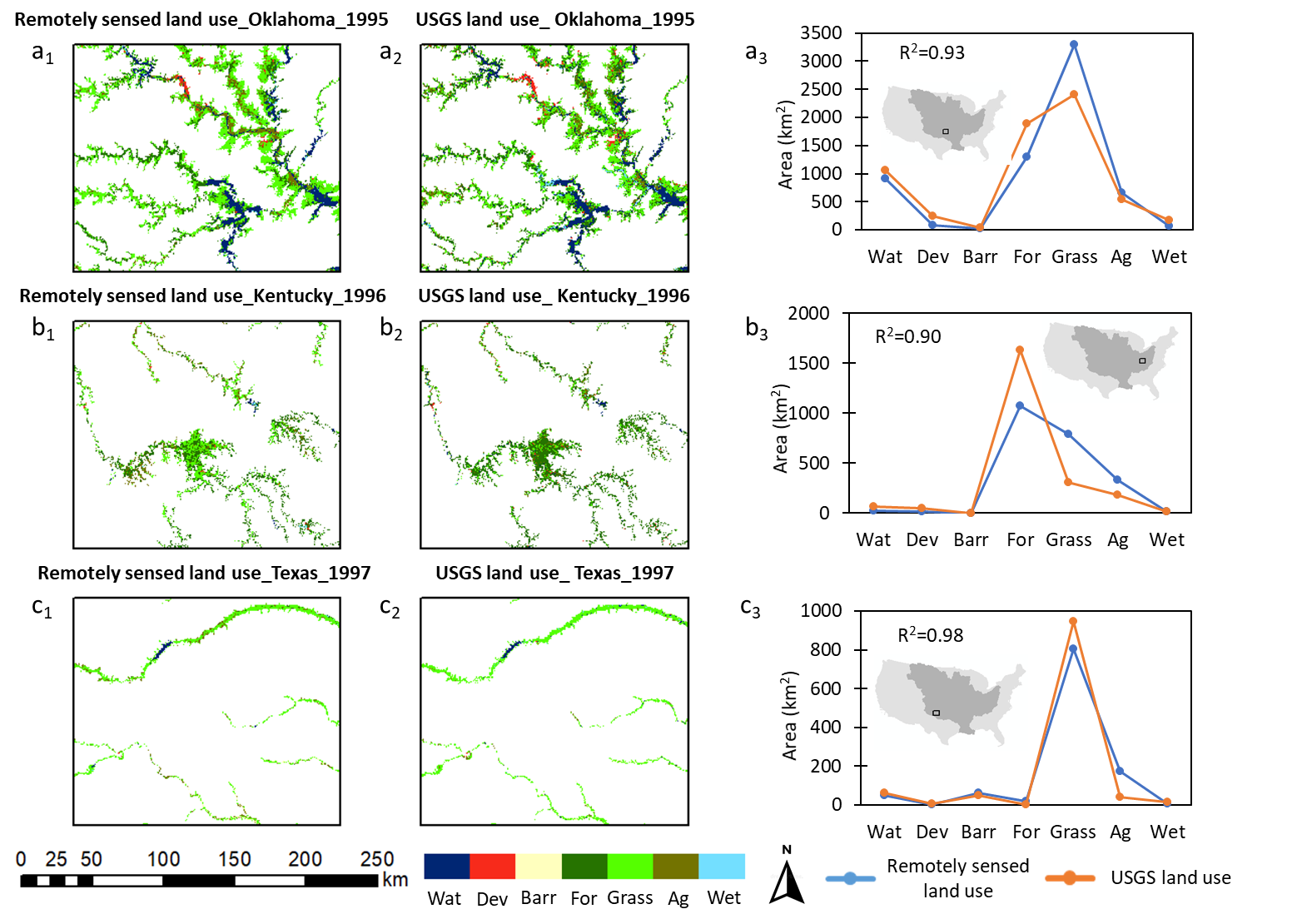


**Supplementary Figure 3.** Spatial comparison between input and reference land use datasets in three different years. The subplots a-c correspond to zoomed-in portions of the Mississippi River Basin floodplains in Oklahoma, Kentucky, and Texas, respectively. a_1_, b_1_, c_1_ show ESA’s Climate Change Initiative (CCI) land use maps based on satellite observations (hereafter, the remotely sensed land use)^3,4^, while a_2_, b_2_, c_2_ show the land use data obtained from USGS land modeling framework^1,2^. The remotely sensed land use (a_1_ - c_1_) was our reference to validate the spatial consistency of the USGS land use (a_2_ – c_2_; the input land use data in our methodology). Subplots a_3_ - c_3_ show the correlation between remotely sensed and USGS datasets across different land use classes within a given spatial domain. The generic land use classes include water, developed, barren, forest, grassland, agriculture, and wetland (abbreviated as Wat, Dev, Barr, For, Grass, Ag, and Wet, respectively).


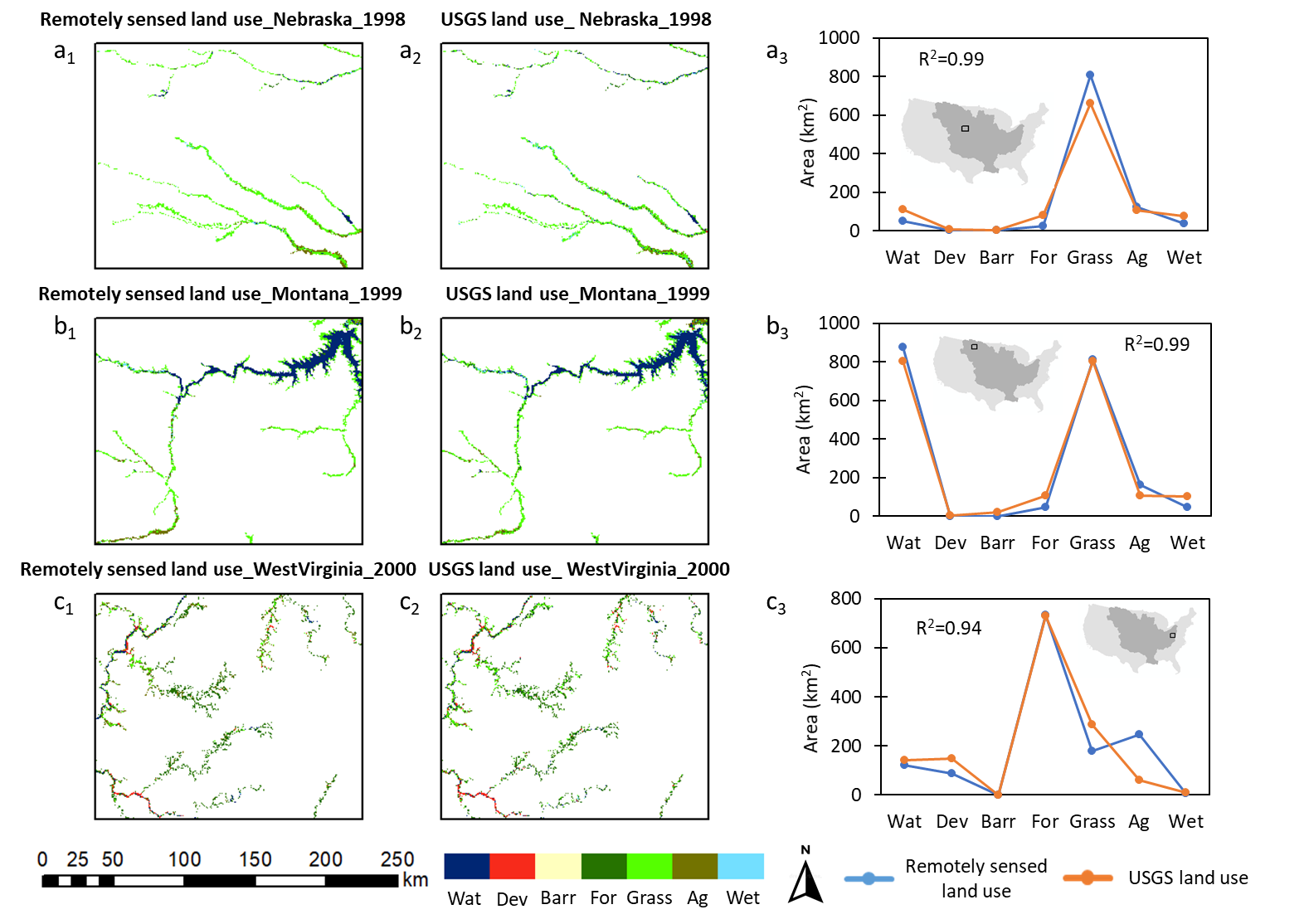


**Supplementary Figure 4.** Spatial comparison between input and reference land use datasets in three different years. The subplots a-c correspond to zoomed-in portions of the Mississippi River Basin floodplains in Nebraska, Montana, and West Virginia, respectively. a_1_, b_1_, c_1_ show ESA’s Climate Change Initiative (CCI) land use maps based on satellite observations (hereafter, the remotely sensed land use)^3,4^, while a_2_, b_2_, c_2_ show the land use data obtained from USGS land modeling framework^1,2^. The remotely sensed land use (a_1_ - c_1_) was our reference to validate the spatial consistency of the USGS land use (a_2_ – c_2_; the input land use data in our methodology). Subplots a_3_ - c_3_ show the correlation between remotely sensed and USGS datasets across different land use classes within a given spatial domain. The generic land use classes include water, developed, barren, forest, grassland, agriculture, and wetland (abbreviated as Wat, Dev, Barr, For, Grass, Ag, and Wet, respectively).

# **References**

1. Sohl, T.L., Reker, R.R., Bouchard, M.A. et. al., 2018a. Modeled historical land use and land cover for the conterminous United States: 1938-1992. *U.S. Geological Survey data release,* <https://doi.org/10.5066/F7KK99RR>
2. Sohl, T.L., Sayler, K.L., Bouchard, M.A. et al, 2018b. Conterminous United States Land Cover Projections - 1992 to 2100. *U.S. Geological Survey data release,* <https://doi.org/10.5066/P95AK9HP>
3. Climate Change Initiative (CCI) of the European Space Agency, 2018. *CCI-LC products.* <http://maps.elie.ucl.ac.be/CCI/viewer/download.php>
4. European Space Agency. 2017. *Land Cover CCI Product User Guide Version 2. Tech. Rep.* <http://maps.elie.ucl.ac.be/CCI/viewer/download/ESACCI-LC-Ph2-PUGv2_2.0.pdf>.
